# Editorial: Grief Disorders: Clinical, Cultural, and Epidemiological Aspects

**DOI:** 10.3389/fpsyt.2021.681523

**Published:** 2021-05-20

**Authors:** Geert E. Smid, Clare Killikelly, Birgit Wagner

**Affiliations:** ^1^ARQ National Psychotrauma Centre, Diemen, Netherlands; ^2^Department of Humanist Chaplaincy Studies, University of Humanistic Studies, Utrecht, Netherlands; ^3^Department of Psychology, University of Zurich, Zurich, Switzerland; ^4^Medical School Berlin, Berlin, Germany

**Keywords:** prolonged grief disorder, persistent complex bereavement disorder, risk, meaning attribution, epidemiology, cultural aspects

Grief disorders have recently been included in international diagnostic classifications, including the 5th edition of the Diagnostic and Statistical Manual of Mental Disorders [DSM-5; (1)] and the 11th edition of the International Classification of Diseases [ICD-11; (2)]. Specifically, DSM-5 now includes the category “other specified trauma- and stressor-related disorder,” with *persistent complex bereavement disorder* as one of the specific examples, and ICD-11 includes *prolonged grief disorder* (PGD). Importantly, the DSM-5 steering committee has announced an update to DSM-5 to include PGD as a separate disorder (3).

The inclusion of grief disorders marks international recognition of the clinical relevance of grief disorders and has prompted mental health professionals to diagnose and treat disordered grief. Although bereavement is known to be capable of precipitating common mental disorders such as depression and posttraumatic stress disorder, to many clinicians, the concept of a grief disorder is new and therefore unfamiliar. This Research Topic contributes toward filling the knowledge gap surrounding disordered grief by highlighting clinical, cultural, and epidemiological aspects of grief disorders.

While clinical aspects include diagnosis, prevention, and treatment of grief disorders, cultural aspects involve the provision of care in a context of multiculturalism and globalization. Epidemiological aspects encompass both clinical and cultural aspects and include risk for the development or maintenance of disordered grief as well as factors that contribute to resilience and recovery. Risk and resilience factors of grief disorders relate to meaning attribution following loss and can be divided into five categories (4). First, *loss-related factors* include non-natural or sudden causes of death and the person's immediate grief response. Second, *cultural factors* include beliefs, rituals, and care provision. Third, *social factors* include social support, juridical, political, and economic factors, among others. Fourth, *individual factors* include gender and age, neurobiological and immunological correlates, personality and attachment, and history of trauma and loss. Fifth, *factors related to the relationship with the deceased* include a variety of factors, such as the nature and quality of the relationship, farewell rituals, and involvement in the death. Figure 1 presents an overview of potential risk and resilience factors.

**Figure 1:**
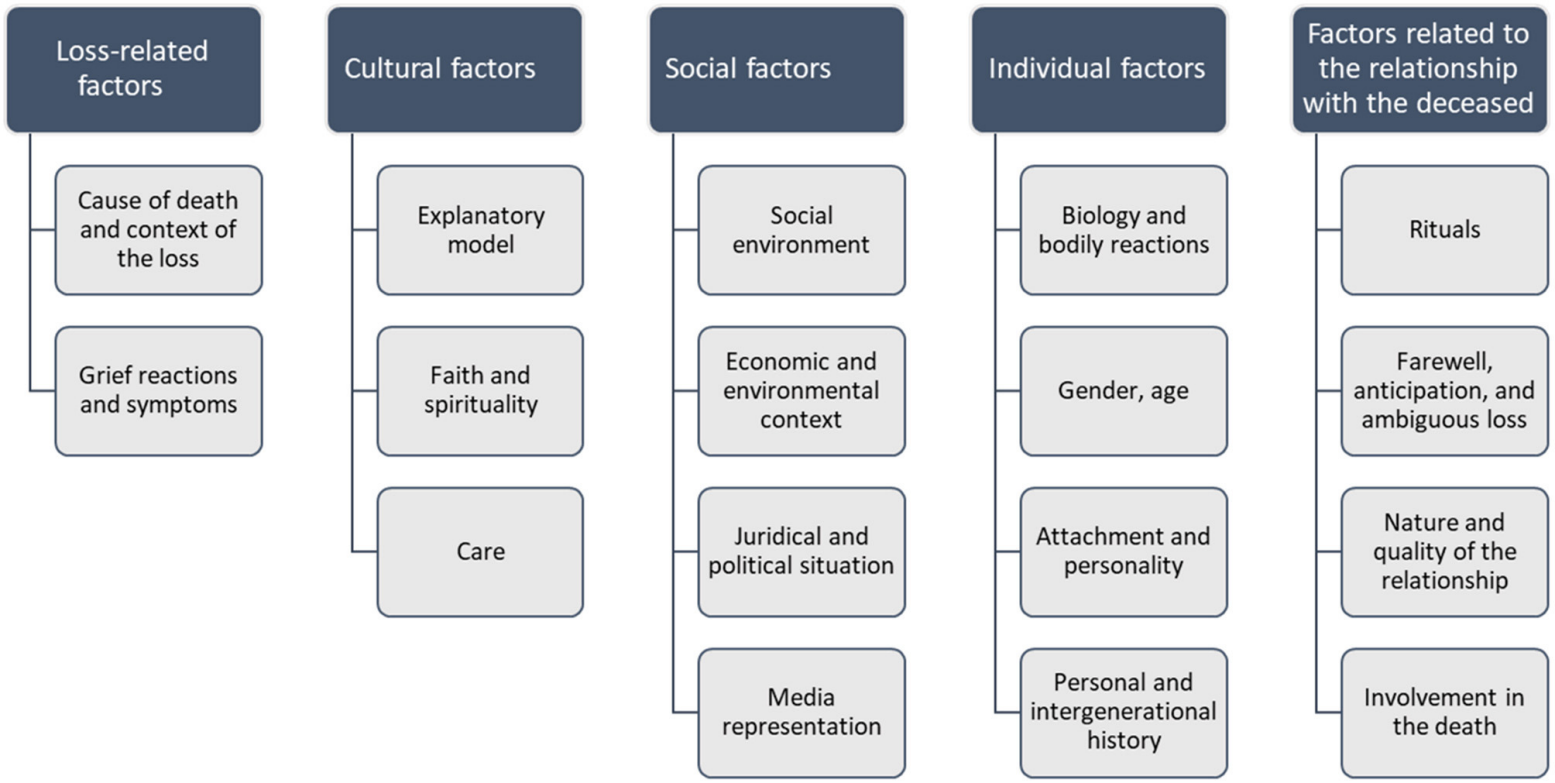
Grief disorders: overview of potential risk and resilience factors.

The breadth of these factors highlights the profound implications the inclusion of grief disorders has for the organization and provision of mental health care, spiritual care, and end-of-life care in the prevention and treatment of grief disorders. Beyond care provision, the support from relatives, collective rituals, economic compensation and other circumstances that are shaped collectively may play a role in enhancing societal resilience and preventing disordered grief. The articles included in this collection address many of these aspects, providing essential insights while emphasizing the need for further scientific enquiry.

## Clinical Aspects: Diagnosis

O'Connor et al. investigated the availability of valid questionnaires, interviews, and cutoffs for ICD-11 PGD. The present systematic review reveals that no such valid tools are available for ICD-11 PGD or any of the earlier diagnostic constructs for PGD. They call for action in the field of bereavement research to start developing these tools now. Eisma et al. propose to conduct multiverse analysis on existing data, by applying different sets of criteria and diagnostic rules to determine how these affect the prevalence and other characteristics of ICD-11 PGD. After reviewing pioneering papers applying multiverse analyses to ICD-11 PGD, they recommend systematic application of multiverse analyses to help define current criteria for ICD-11 PGD, and future versions of this diagnosis.

Sveen et al. examined psychometric properties of the Swedish PG-13 as well as the latent structure of prolonged grief using the PG-13. Their findings support the use of the Swedish PG-13 to measure prolonged grief in bereaved adults. The latent structure of prolonged grief comprised two or three factors, and not a unidimensional structure as suggested previously.

Nielsen et al. used prospective data pre- and post-bereavement to explore functional impairment in PGD (assessed using the PG-13). They found that one in five bereaved relatives still reported overall functional impairment at 3 years after bereavement, although impairment in daily activities and social functioning improved from before to after bereavement. Also, functional impairment prior to bereavement was associated with the development of PGD. Finally, shedding light on the debate whether clinicians should consider mania as a possible bereavement reaction (Carmassi et al.), reviewed cases demonstrating a possible relationship between bereavement and a first manic episode.

## Clinical Aspects: Treatment

Research on various grief-focused treatments informs clinical practice. Rubin et al. describe the two-track model of bereavement, comprising biopsychosocial functioning and relationship to the deceased. Their case illustration of a woman who discovered her husband's dead body after his suicide illustrates how the assessment and intervention program was informed by the two tracks included in the model.

Soydas et al. use self-reported data on symptoms of posttraumatic stress, prolonged grief, depression, anxiety, and functional impairment from 929 homicidally bereaved, treatment receiving adults. They explore whether the observed effects of the intervention provided as part of the UK National Homicide Bereavement Service are associated with sociodemographic, homicide-related and clinical characteristics. Symptom reduction and improvements in functional impairment during treatment were negatively influenced by having a history of mental illness. A slow course of justice or the absence of a verdict negatively impacted treatment response on some of the observed outcomes.

Vogel et al. evaluate the feasibility and the treatment effects of present-centered therapy (PCT) in a small sample of adults with PGD. Large decreases in PGD symptom severity at posttreatment and 3-month follow-up assessment as well as significant decreases in self-reported PGD symptoms, depression, and general psychological distress were observed. They conclude that PCT is a promising treatment for PGD and may serve as a strong comparator for future studies evaluating the efficacy of certain treatment elements in patients suffering from PGD.

Wagner et al. investigate the effectiveness of web-based bereavement interventions compared with control groups in reducing symptoms of grief in adults. The results of this meta-analysis suggest that internet-based treatments, based on CBT, can help to reduce the symptoms related to the loss of a significant person.

## Cultural Aspects

Stelzer et al. summarize key findings from East-Asian research groups that challenge the applicability of North American and European definitions of PGD. This review finds that this area is currently facing a crisis in terms of the heterogenic use of different diagnostic criteria sets yielding widely different prevalence rates. It is important for researchers and clinicians to share knowledge and develop a consensus on required cultural caveats and culturally specific symptoms to improve assessment and treatment of disordered grief.

Djelantik et al. show that although refugees experience multiple post-migration stressors, they report significant symptom reductions during traumatic grief focused treatment. However, undocumented asylum seekers were more likely to drop out before completion of the treatment. Ongoing conflict in the country of origin and the total number of post-migration stressors were associated with smaller symptom reductions. They recommend that clinicians should recognize the effects of post-migration stressors on the course of symptoms and educate their refugee patients to support realistic treatment expectations.

Lacour et al. explore the relationship between the severity of PGD and cognitive and emotional factors, potentially traumatic events and living difficulties after arrival in the host country, in a clinical sample of refugees. Their findings suggest that self-efficacy and emotion regulation are determinants of PGD and constitute potential targets for treatment interventions.

Stammel et al. investigate associations between attitudes toward reconciliation and prolonged grief (PG) in bereaved survivors of the Khmer Rouge regime in Cambodia 30 years after the end of the regime. They show that a higher PG-symptom severity was related to less openness toward reconciliation. These findings are discussed in relation to cultural and religious views on death and mourning in Cambodia. The results of the study underline the importance of considering PG in regard to reconciliation efforts in post-conflict regions.

## Epidemiological Aspects

Due to the Covid-19 pandemic, grief disorders will likely become a worldwide public health concern. Global death rates due to Covid-19 now exceed 1.5 million and the end of the pandemic is not yet in sight. Gesi et al. draw attention to people at risk of developing grief disorders due to the Covid-19 crisis, providing a useful framework for the management of bereaved people during the acute phase of the pandemic and for the implementation of tailored services in the future.

Fisher et al. investigated coping strategies in surviving relatives of U.S. military service members who died by combat, accident, or suicide. They determined that there are differential contributions of coping strategies to grief severity, depression and posttraumatic growth. Avoidant coping was a potent contributor to negative outcomes and inhibitor of posttraumatic growth. Although considering the possibility of death appeared to mitigate negative outcomes among relatives who survived combat death, avoidance of that possibility is likely protective for the majority of family members whose loved ones return home safely.

In a prospective cohort study, Kristensen et al. show that most bereaved family members following terror display a long-lasting and difficult grieving process, and that comorbid PTSD symptoms contribute to the slow recovery over the first 3 years after the incident. This has important implications for the psychosocial follow-up, which should highlight both the need for early intervention and long-term measures. Also, adequate skills among clinicians in screening for and treating both prolonged grief and posttraumatic stress is needed.

## Author Contributions

GS: wrote the first draft of the manuscript. All authors contributed to manuscript revision, read, and approved the submitted version.

## Conflict of Interest

The authors declare that the research was conducted in the absence of any commercial or financial relationships that could be construed as a potential conflict of interest.
